# Vertical transmission of naturally occurring Bunyamwera and insect-specific flavivirus infections in mosquitoes from islands and mainland shores of Lakes Victoria and Baringo in Kenya

**DOI:** 10.1371/journal.pntd.0006949

**Published:** 2018-11-19

**Authors:** Yvonne Ukamaka Ajamma, Thomas Ogao Onchuru, Daniel O. Ouso, David Omondi, Daniel K. Masiga, Jandouwe Villinger

**Affiliations:** 1 International Centre of Insect Physiology and Ecology (*icipe*), Nairobi, Kenya; 2 Department of Evolutionary Ecology, Institute of Organismic and Molecular Evolution, Johannes Gutenberg University, Mainz, Germany; 3 Biochemistry and Molecular Biology Department, Egerton University, Egerton, Kenya; INDEPENDENT RESEARCHER, UNITED STATES

## Abstract

**Background:**

Many arboviruses transmitted by mosquitoes have been implicated as causative agents of both human and animal illnesses in East Africa. Although epidemics of arboviral emerging infectious diseases have risen in frequency in recent years, the extent to which mosquitoes maintain pathogens in circulation during inter-epidemic periods is still poorly understood. This study aimed to investigate whether arboviruses may be maintained by vertical transmission via immature life stages of different mosquito vector species.

**Methodology:**

We collected immature mosquitoes (egg, larva, pupa) on the shores and islands of Lake Baringo and Lake Victoria in western Kenya and reared them to adults. Mosquito pools (≤25 specimens/pool) of each species were screened for mosquito-borne viruses by high-resolution melting analysis and sequencing of multiplex PCR products of genus-specific primers (alphaviruses, flaviviruses, phleboviruses and Bunyamwera-group orthobunyaviruses). We further confirmed positive samples by culturing in baby hamster kidney and *Aedes* mosquito cell lines and re-sequencing.

**Principal findings:**

*Culex univittatus* (2/31pools) and *Anopheles gambiae* (1/77 pools) from the Lake Victoria region were positive for Bunyamwera virus, a pathogenic virus that is of public health concern. In addition, *Aedes aegypti* (3/50), *Aedes luteocephalus* (3/13), *Aedes* spp. (2/15), and *Culex pipiens* (1/140) pools were positive for Aedes flaviviruses at Lake Victoria, whereas at Lake Baringo, three pools of *An*. *gambiae* mosquitoes were positive for Anopheles flavivirus. These insect-specific flaviviruses (ISFVs), which are presumably non-pathogenic to vertebrates, were found in known medically important arbovirus and malaria vectors.

**Conclusions:**

Our results suggest that not only ISFVs, but also a pathogenic arbovirus, are naturally maintained within mosquito populations by vertical transmission, even in the absence of vertebrate hosts. Therefore, virus and vector surveillance, even during inter-epidemics, and the study of vector-arbovirus-ISFV interactions, may aid in identifying arbovirus transmission risks, with the potential to inform control strategies that lead to disease prevention.

## Author summary

The East African region is endemic to diverse mosquito-transmitted arboviruses, though little is known about the role of vertical transmission in maintaining these viruses within mosquito vector populations during inter-epidemic periods. We sampled mosquito larvae from the Lake Baringo and Lake Victoria regions of Kenya and reared them to adults in the laboratory before screening them for mosquito-associated viruses by multiplex RT-PCR-HRM, cell culture, and sequencing. From the Lake Victoria region, we detected the arbovirus, Bunyamwera, which can cause febrile illness in humans, in *Culex univittatus* and vector competent *Anopheles gambiae* mosquitoes. We also identified diverse insect-specific flaviviruses in *Aedes aegypti*, *Aedes luteocephalus*, *Aedes* spp. and *Culex pipiens* mosquitoes. From the Lake Baringo region, we detected Anopheles flavivirus in *An*. *gambiae* mosquitoes. These findings demonstrate that naturally occurring vertical transmission potentially maintains viruses in circulation within the sampled vector species populations. Therefore, mosquitoes may potentially transmit a pathogenic arbovirus during their first bite after emergence. Because various insect-specific flaviviruses have recently been found to either inhibit or enhance replication of specific arboviruses in mosquitoes, their vertical transmission, as observed in this study, has implications as to their potential impact on both horizontal and vertical transmission of medically important arboviruses.

## Introduction

The East African Great Lakes region is a recognized hotspot for a broad diversity of arthropod-borne viruses (arboviruses) [1] that affect humans and animals [2] and are transmitted by several mosquito genera (mostly *Culex* Linnaeus, *Aedes* Meigen, *Anopheles* Meigen, *Mansonia* Blanchard, and *Aedeomyia* Theobald species) [3–5]. Some mosquito species are capable of naturally maintaining viruses in circulation through vertical transmission [6–9]–up to 38 generations for San Angelo (SA) virus in *Aedes albopictus*, though with progressive decline in filial infection rate (FIR) in laboratory population bottlenecks [10].

The Lake Victoria and Lake Baringo regions of Kenya have historically been associated with arboviral diseases [11] and have unique lake and island biogeographies [12] in which arboviruses exist [5]. Outbreaks in the 1960s around the Lake Victoria basin involved Semliki Forest, chikungunya, and o’nyong-nyong viruses that are vectored by *Culex*, *Aedes*, and *Anopheles* mosquito species, respectively [13]. More recent studies have found seropositivity for arboviruses in humans [14–16]. During the recent 2006–2007 Rift Valley fever (RVF) outbreak in Baringo County, 10 mosquito species were implicated as potential vectors, among which *Aedes pembaensis* Theobald, *Culex univittatus* Theobald, and *Culex bitaeniorhynchus* Giles were reported as potential vectors for the first time [11].

Although widespread arboviral activity in human populations has been documented in the Lake Victoria and Lake Baringo basins, the role of vertical transmission among mosquito vectors in the maintenance of arboviruses within ecologies remains poorly understood [17]. To ascertain the competence of mosquitoes to horizontally transmit arboviruses between hosts, many methods have been used to collect and test different mosquito body parts (abdomen, saliva, and legs) for arboviruses [18]. However, vertical transmission of arboviruses from adult female mosquitoes to their offspring can also maintain viruses in circulation for generations within mosquito populations [6–10]. To investigate how vertical transmission in different mosquito species in Homa Bay and Baringo counties of Kenya may be maintaining endemic arboviruses in circulation, we set out to identify arboviral infections in laboratory-reared adults of field-caught larvae and pupae.

## Methods

### Study area, mosquito sampling and rearing

**Fig 1:**
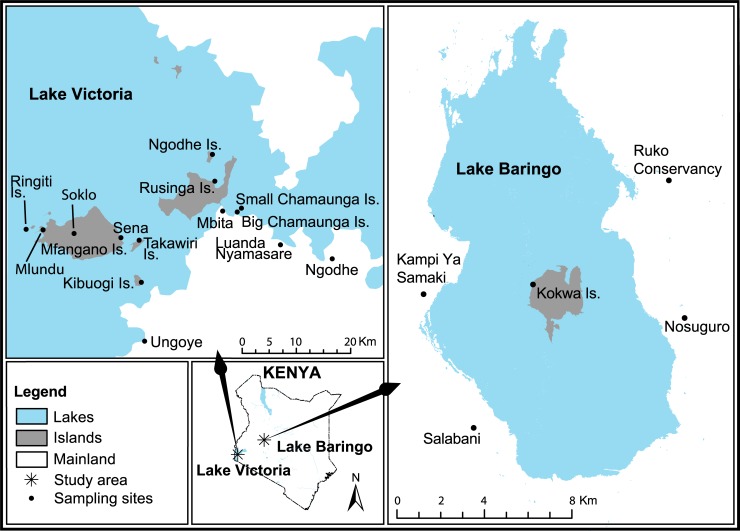
Map of the study areas in and around Lake Baringo along the Great Rift Valley and Lake Victoria on the western part of Kenya.

In 2012, immature mosquitoes were sampled from islands and mainland shores of Lake Baringo (in Baringo County along the Great Rift Valley) and Lake Victoria (in Homa Bay County) of Kenya (Fig 1) during the rainy season. In Baringo County, samples were collected in July and October 2012 from Kokwa Island, Nosuguro, Salabani, Kampi ya Samaki, Sirata, and Ruko. In Homa Bay County, samples were collected in April, May and November 2012 from Ringiti, Chamaunga, Kibuogi, Rusinga, Takawiri, Mfangano and Ngodhe Islands, and Ungoye, Luanda Nyamasare, Mbita and Ngodhe mainland sites on the Kenyan part of Lake Victoria. Sampling was conducted on unprotected public land concurrently with an adult mosquito genetic diversity survey conducted in the same study areas [19].

We collected eggs, larvae, and pupae with 350-ml standard dippers (Bioquip Products, USA) from their breeding sites and transported them to the Martin Lüscher Emerging Infectious Disease (ML-EID) Laboratory at the Duduville campus of the International Centre of Insect Physiology and Ecology (*icipe*) in Nairobi, Kenya. In the laboratory, we reared them to adults in their field-collected breeding water at 28°C temperature, 80% relative humidity, and 12-hour day and night cycles [20,21].

### Ethics statement

Before sampling, we obtained ethical clearance for the study from the Kenya Medical Research Institute (KEMRI) ethics review committee (Approval Ref: Non-SSC Protocol #310) and no protected species were sampled.

### Mosquito morphological identification and homogenization

All reared adult mosquitoes were identified and sorted using morphological keys [22–25] in petri-dishes on frozen ice packs to keep them cold and to avoid degradation of any viruses in the samples. The ice packs were wrapped with paper towels to absorb moisture and prevent frosting of the petri-dishes. We stored pools of ≤25 reared adult mosquitoes in well-labelled 1.5 ml microcentrifuge tubes according to species, larval collection sites, sex, and dates in tubes in a -80°C freezer.

Ten pieces of 2.0-mm yttria-stabilized zirconia beads (Glen Mills, Clifton, NJ) and 400 μl of cold homogenization media (2% L-glutamine, 15% fetal bovine serum) (Sigma-Aldrich, St. Louis, USA) were added to each tube, which were placed on ice to keep them cold. The mosquito pools were then homogenized for 10 seconds in Mini-BeadBeater-16 (BioSpec, Bartlesville, OK, USA) followed by centrifugation for 10 seconds in a bench top centrifuge (Eppendorf, USA) at 1,500 relative centrifugal force (rcf) and 4°C. Aliquots of 210 μl of each homogenate were used for nucleic acid extraction and the remaining aliquots were stored in -80°C freezer as stock.

### Detection and identification of viruses in adult mosquitoes reared from immatures

Nucleic acid (NA) was extracted from the 210-μl mosquito homogenate aliquots using the MagNA 96 Pure DNA and Viral NA Small Volume Kit (Roche Applied Science, Penzberg, Germany) in a MagNA Pure 96 automatic extractor (Roche Applied Science) and eluted into a final volume of 50 μl according to the manufacturer’s instructions. A reverse transcription-multiplex polymerase chain reaction with high-resolution melting (RT-PCR-HRM) analysis based arbovirus screening protocol recently developed by Villinger et al. [26] was used to rapidly screen many samples and detect the presence of four arbovirus genera, namely, *Alphavirus* (family Togaviridae), *Flavivirus* (family Flaviviridae), Bunyamwera-group *Orthobunyavirus* (family Peribunyaviridae), and *Phlebovirus* (family Phenuiviridae). Briefly, the High Capacity cDNA Reverse Transcription (RT) kit (Life Technologies, USA) was used to synthesize complimentary DNA (cDNA) of the nucleic acid extracts. cDNA synthesis from 5 μl of extracted nucleic acids was performed in 10-μl reaction volumes with final concentrations of 1x RT Buffer, 4 mM dNTP mix, 2.5 U/μl MultiScribe Reverse Transcriptase, 1 U/μl RNase Inhibitor, 600 μM non-ribosomal random hexanucleotide primers [27]. Reverse transcriptions were performed in a Veriti 96-Well Thermal Cycler (Applied Biosystems, Singapore) at 25°C for 10 minutes, 37°C for 2 hours, 85°C for 5 minutes and held at 4°C.

We used established multiplex RT-PCR thermocycling conditions [26] in a HRM capable Rotor-Gene Q real-time PCR thermocycler (Qiagen, Redwood city, CA, USA) to screen for virus sequences in cDNA templates. Ten microliter reactions consisting of 1 μl cDNA template, 5 μl 2x MyTaq HS Mix (Bioline, UK), 1 μl of 50 μM SYTO-9 saturating intercalating dye (Life Technologies), and multiplex PCR primers at concentrations given in Table 1. The QIAgility robot (Qiagen) for liquid handling was used to set up the reaction mixture. Touchdown PCR cycling conditions as detailed by Villinger et al. [26] included an initial denaturation at 95°C for 5 minutes, followed by 50 cycles of denaturation at 94°C for 20 seconds, annealing at 63.5–47.5°C for 20 seconds, and extension at 72°C for 5–30 seconds, followed by a final extension at 72°C for 3 minutes. Immediately after PCR, the product was held at 40°C for 1 minute before HRM analyses of PCR product double stranded DNA stability by measuring SYTO-9 fluorescence at 0.1°C temperature intervals increasing every 2 seconds from 75°C to 90°C. PCR grade water was used as negative control, and Bunyamwera (*Orthobunyavirus*), dengue and West Nile (*Flavivirus*), sindbis and Middelburg (*Alphavirus*), and Rift Valley fever (*Phlebovirus*) viruses were used as positive controls. Positive samples were re-run in singleplex reactions (using primers from only one genus; Table 1). Amplicons from singleplex runs were purified with ExoSAP-IT for PCR Product Kit (Affymetrix Inc., USA) and Sanger-sequenced at Macrogen (Korea). Samples that were positive for the *Flavivirus* genus by HRM analysis were further sequenced from nested PCR products using the 2NS5F (5’-GCNATNTGGTWYATGTGG-3’) and 2NS5Re (5’-TRTCTTCNGTNGTCATCC-3’) primers that amplify longer nucleotide fragments (~930 nt) of *Flavivirus* NS5 genes [28]. Resulting nucleotide sequences were edited using Geneious R7.1.9 software (created by Biomatters) [29].

**Table 1:** Multiplex primers used for virus RNA identification.

Target virus genus	Primer name	Primer Sequence (5'-3’)	Reaction concentration (nM)	Reference
Bunyamwera group *Orthobunyavirus*	Bunya group F	CTGCTAACACCAGCAGTACTTTTGAC	167	[30]
	Bunya group R	TGGAGGGTAAGACCATCGTCAGGAACTG	167	
*Phlebovirus*	Phlebo JV3a F	AGTTTGCTTATCAAGGGTTTGATGC	500	[26]
	Phlebo JV3b F	GAGTTTGCTTATCAAGGGTTTGACC	500	
	Phlebo JV3 R	CCGGCAAAGCTGGGGTGCAT	500	[26]
*Alphavirus*	Vir 2052 F	TGGCGCTATGATGAAATCTGGAATGTT	400	[31]
	Vir 2052 R	TACGATGTTGTCGTCGCCGATGAA	400	
*Flavivirus*	Flavi JV2a F	AGYMGHGCCATHTGGTWCATGTGG	200	[26]
	Flavi JV2b F	AGCCGYGCCATHTGGTATATGTGG	125	
	Flavi JV2c F	AGYCGMGCAATHTGGTACATGTGG	125	
	Flavi JV2d F	AGTAGAGCTATATGGTACATGTGG	50	
	Flavi JV2a R	GTRTCCCADCCDGCDGTRTCATC	400	
	Flavi JV2b R	GTRTCCCAKCCWGCTGTGTCGTC	100	

### Virus culture

To validate that the sequenced targets were truly viral and not viral genome segment inserts in the mosquito genome, a fraction of the original mosquito homogenates that were PCR-positive for potential arboviruses were subjected to cell culture in vertebrate BHK-21 (Kidney of Syrian hamster, Lot: 59300875 from ATCC) and *Ae*. *albopictus* clone C6/36 (Whole larva of Asian tiger mosquito, Lot: 60400699 from ATTC) cell lines. Stock mosquito homogenates of 19 samples with sequences that aligned with known viruses on GenBank [32] and RNA virus databases were subjected to cell culture. The homogenates were thawed on ice and clarified by centrifugation at 15,000 rcf and 4°C in a bench top centrifuge (Eppendorf 5417R) for 5 minutes. One hundred microlitres of the clarified supernatant were aseptically inoculated in each of sub-confluent BHK-21 and C6/36 cell lines in a 24-well culture plate. The BHK-21 cells were initially aseptically grown in growth media (GM; pH 7.5) made of 2% Minimum Essential Media (MEM; +Eagle’s salt, +25 Mm HEPES) with 10% FBS, 2% L-glutamine and 1% antimycotic (Sigma-Aldrich). The C6/36 GM contained same proportions of respective constituents as the BHK-21 GM, but with the addition of 1% non-essential amino acids (GIBCO, UK). The inoculated cell lines were incubated for 14 days and observed daily for any change in the morphology of the cell line caused by viral infection, also known as the cytopathic effect (CPE). Virus presence was ascertained as CPE. During the initial 14-day incubation period, any contaminated cell culture was purified using a 0.22 μm syringe filter [33] and re-tested. Further, RNA was extracted from cell culture wells that showed CPE and tested in single-genus arbovirus RT-PCR-HRM reactions and re-confirmed by sequencing, as described above.

### Phylogenetic sequence analysis

Using Basic Local Alignment Search Tool (BLAST) [34], initial searches were performed for comparison of all obtained virus sequences with those in GenBank. This was followed by sequence alignments using the default settings of the MAFFT v7.017 [35] plugin in Geneious software, to identify virus segments. Maximum likelihood phylogenetic relationships of the study’s insect-specific flaviviruses (ISFVs) NS5 sequences with those of related ISFVs were analyzed using PhyML version 3.0 [36], employing the Akaike information criterion [37] for automatic selection of the general time reversible (GTR) sequence evolution model. Tree topologies were estimated using nearest neighbour interchange (NNI) improvements over 1000 bootstrap replicates. Rooting the phylogeny to the yellow fever vaccine strain sequence (GenBank accession NC_002031) as an outgroup, the phylogenetic tree was depicted using FIGTREE version 1.4.2 [38].

## Results

**Fig 2:**
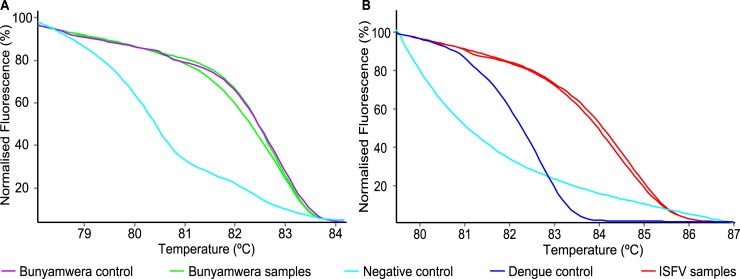
Virus HRM profiles from mosquito pools positive for (A) Bunyamwera virus and (B) insect-specific flaviviruses (ISFVs).

A total of 4,453 adult mosquitoes comprised of nine *Aedes*, six *Anopheles*, 16 *Culex* and one *Mimomyia* species were reared from immatures (Table 2). Among 612 pools of ≤25 mosquito samples per pool, 92 pools were from Baringo County and 520 pools were from Homa Bay County. Among mosquito pools from 32 species sampled in Homa Bay County, Bunyamwera virus (*Orthobunyavirus*) was the only vertically transmitted arbovirus (pathogenic to vertebrates) detected. It was identified by HRM analysis (Fig 2A), culture, and DNA sequencing (143 nt; 100% identity to GenBank accession KM507344, S1 Fig) from female *Anopheles gambiae* from Luanda Nyamasare (1/77 pools) and *Cx*. *univittatus* from Rusinga (2/31 pools) (Table 2) that were reared from larvae sampled in November 2012. However, no vertically transmitted pathogenic arbovirus was detected in Baringo County samples.

**Table 2:** Viruses identified from pools of reared mosquito species from Baringo and Homa Bay Counties of Kenya.

Mosquito species	Grand Total	Lake Baringo (Baringo County)			Lake Victoria (Homa Bay County)		
		Number of mosquitoes	Number of pools	Virus positive pools	Number of mosquitoes	Number of pools	Virus positive pools
*Ae*. *hirsutus*	5	0	-	-	5	3	-
*Ae*. *furcifer*	3	0	-	-	3	2	-
***Ae*. *aegypti***	729	0	-	-	729	50	**3-CFAV**
*Ae*. *cumminsi*	17	0	-	-	17	1	-
*Ae*. *dentatus*	1	0	-	-	1	1	-
***Ae*. *luteocephalus***	68	0	-	-	68	13	**3-AeFV**
*Ae*. *metallicus*	275	0	-	-	275	22	-
*Ae*. *ochraceus*	1	0	-	-	1	1	-
*Ae*. *vittatus*	301	0	-	-	301	20	-
***Aedes* spp.**	185	0	-	-	185	15	**1-AeFV, 1-CFAV**
*An*. *coustani*	1	0	-	-	1	1	-
*An*. *funestus*	3	0	-	-	3	3	-
***An*. *gambiae***	300	35	15	**3-An(g)FV**	265	77	**1-BUN**
*An*. *pharoensis*	6	6	3	-	0	-	-
*An*. *rufipes*	1	0	-	-	1	1	-
*An*. *ziemanni*	2	0	-	-	2	2	-
*Cx*. *annulioris*	4	0	-	-	4	1	-
*Cx*. *duttoni*	15	0	-	-	15	4	-
*Cx*. *neavei*	26	9	3	-	17	6	-
***Cx*. *pipiens***	1732	337	32	-	1395	140	**1-AeFV**
*Cx*. *poicilipes*	87	3	2	-	84	12	-
*Cx*. *simpsoni*	24	0	-	-	24	15	-
*Cx*. *striatipes*	2	0	-	-	2	1	-
*Cx*. *terzii*	2	0	-	-	2	2	-
*Cx*. *theileri*	1	0	-	-	1	1	-
***Cx*. *univittatus***	100	37	15	-	63	31	**2-BUN**
*Cx*. *vansomereni*	10	0	-	-	10	4	-
*Cx*. *watti*	44	0	-	-	44	15	-
*Cx*. *zombaensis*	19	5	3	-	14	3	-
*Cx*. *tigripes*	24	0	-	-	24	15	-
*Cx*. *adersianus*	8	0	-	-	8	2	-
*Cx*. *rima*	20	0	-	-	20	2	-
*Culex* spp.	427	18	10	-	409	53	-
*Mi*. *splendens*	10	9	9	-	1	1	-
Total	4453	459	92	3	3994	520	12
Species positive	5			1			5

BUN is Bunyamwera virus; An(g)FV is Anopheles gambiae flavivirus; AeFV is Aedes flavivirus; CFAV is cell fusing agent virus. *Aedes* spp. and *Culex* spp. are mosquito specimens that could only be identified to genus.

**Fig 3:**
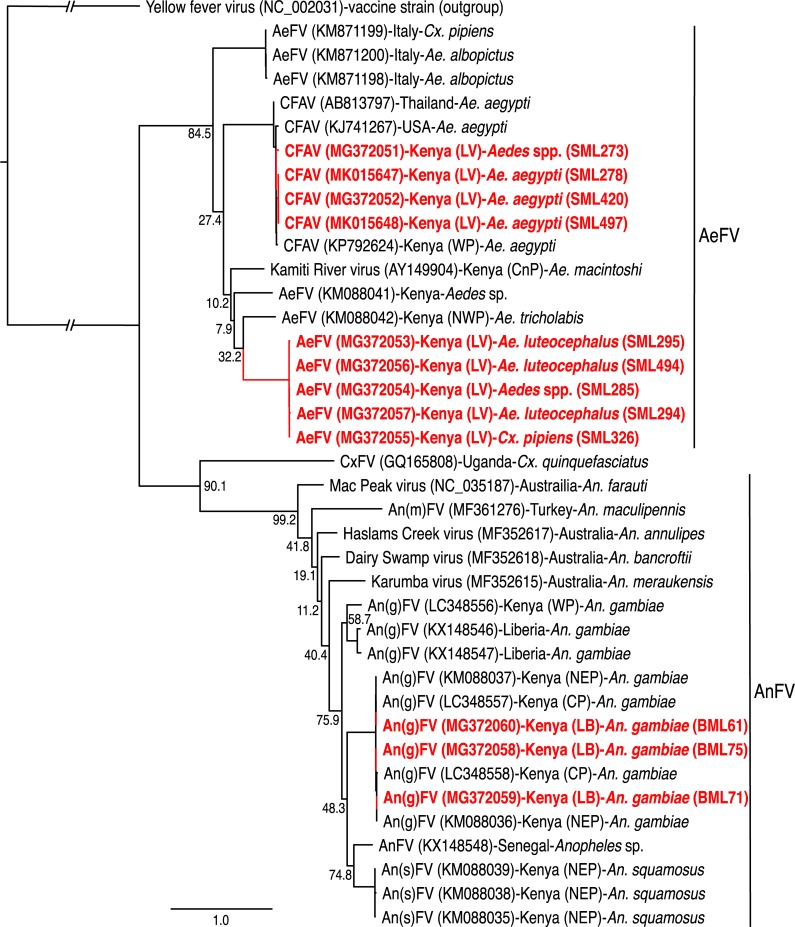
PhyML tree of insect-specific flavivirus NS5 gene sequences associated with mosquito pools from Lake Baringo and Lake Victoria. The phylogeny was created from 779–908 nt fragments. GenBank accessions are indicated in parentheses, followed by collection country with province in parentheses, and the mosquito species in which they were identified. Viruses identified from mosquitoes reared from larval collections in this study are indicated in bold with specimen ID’s in brackets. Bootstrap percentages at the major nodes are of agreement among 1000 replicates. The branch length scale represents substitutions per site. The gaps indicated in the branches to the yellow fever virus outgroup represent 3.0 substitutions per site. (LB: Lake Baringo; LV: Lake Victoria; NEP: North-Eastern Province; WP: Western Province; CnP: Central Province; CP: Coastal Province; RVP: Rift Valley Province).

Further, we detected (Fig 2B) and sequenced ISFV NS5 sequences from 12 mosquito pools (Table 2, GenBank accessions: MG372051-MG372060, MK015647-MK015648) among May 2012 collections. Among Baringo samples, we sequenced three ISFVs from female *An*. *gambiae* mosquitoes (3/15 pools; one pool from Ruko and two pools from Kampi ya Samaki) collected in October 2012 that were closely related to Anopheles gambiae flaviviruses (An(g)FV) that were previously detected in mosquitoes sampled from Kenya’s North-Eastern Province [26], as well as Western and Coastal Provinces (Fig 3). Among Homa Bay County samples, we found Aedes flavivirus (AeFV) NS5 sequences in *Ae*. *luteocephalus* (3/13 pools; two pools from Ungoye and one pool from Mbita) and *Aedes* sp. (1/15 pools; from Takawiri Island), as well as in *Cx*. *pipiens* (1/140 pools; Rusinga Island). We also found cell fusing agent virus (CFAV), the first ISFV originally identified in *Ae*. *aegypti* using an *Ae*. *albopictus* cell line (C6/36) [39], among Homa Bay County *Ae*. *aegypti* (3/50 pools; Mfangano Island) and *Aedes* spp. (1/15 pools; from Ungoye) samples.

## Discussion

We identified natural infections of Bunyamwera virus and ISFVs in diverse anopheline and culicine mosquito species reared to adults from field-collected larvae, demonstrating that these viruses persist transstadially through development to adult stages from naturally infected immature life stages. Since vertical transmission was first identified of vesicular stomatitis virus by phlebotomine sandflies [40] followed by La Crosse virus in *Aedes triseriatus* [41, 42], this mode of maintaining arboviruses within ecosystems has been observed in numerous arboviruses of medical importance circulating in East Africa, including West Nile virus by *Culex* and *Aedes* mosquitoes [43–47], Ndumu virus [48] by *Cx*. *pipiens*, and Zika [49,50], dengue [51–53], chikungunya [54], and RVF [8] viruses by *Aedes* mosquitoes [55]. However, how widespread or important this mode of transmission is in natural ecologies remains poorly understood. While we attribute the naturally occurring virus infections that were transstadially transmitted from immature life stages in this study to vertical transmission from their parents, we cannot completely rule out the possibility that the immature mosquitoes were infected with these viruses from viral contamination in their aquatic environment during early development. However, this mode of transmission if far less likely as past studies indicate that such infection of immature mosquitoes requires unrealistically high viral doses in their aquatic environment [56].

We documented the vertical transmission of the *Orthobunyavirus*, Bunyamwera virus, from naturally occurring infections in two mosquito species–*An*. *gambiae* and *Cx*. *univittatus*–the former of which has previously been found to competently transmit Bunyamwera virus during blood-feeding on suckling mice [57]. This is of public health importance and needs to be monitored closely, as Bunyamwera is an important cause of acute febrile illness in humans (Bunyamwera fever) [58] that is able to reassort with closely related arboviruses to form new viruses, such as Ngari virus, which can cause haemorrhagic fever in humans [59]. With the well-established role of vertical transmission in *Ae*. *triseriatus* mosquitoes of the closely related *Orthobunyavirus*, La Crosse virus [60], the potential of Bunyamwera virus to remain in circulation by vertical transmission within mosquito populations in East Africa, highlights the importance of control strategies focused on vectors and the replication of arboviruses within the vector.

Recent laboratory vector competence studies have found that Bunyamwera virus can be competently transmitted by *An*. *gambiae* and *Ae*. *aegypti* mosquitoes [57], and can naturally infect *Aedeomyia africana*, *Anopheles coustani*, and *Mansonia africana* mosquitoes [5]. However, *Culex quinquefasciatus* was found to be refractory to Bunyamwera virus infection experimentally [57]. Our findings demonstrate that Bunyamwera infection persists from larval stages to adults in *Cx*. *univittatus* mosquitoes as well as in Bunyamwera competent *An*. *gambiae*. This expands the mosquito species, and indeed genera, that may play key roles in maintaining Bunyamwera virus in circulation. Though the vectorial competence of *Cx*. *univittatus* to transmit Bunyamwera virus has not been established, the species is thought to prefer birds as a source of bloodmeals [61] and has recently been found to also feed on dogs, donkeys, sheep, and toads [5], as well as humans [62]. Therefore, *Cx*. *univittatus* may have a greater potential for transmitting arboviruses between birds and other vertebrates to humans, in contrast to the more anthropophilic *An*. *gambiae*.

The vertically transmitted ISFVs, AeFV and CFAV, were only detected in samples from the Lake Victoria region, not only in *Aedes* mosquitoes, but also in *Cx*. *pipiens* (AeFV), though we cannot fully rule out accidental *Aedes* mosquito contamination in the *Cx*. *pipiens* sample. While vertical transmission of ISFVs has been reported experimentally [63–66], which may be as high as 90% [67], this study corroborates its occurrence in natural ecologies [64,65,68,69]. Although ISFVs do not infect mammals and generally have been found to cluster within distinct phylogenetic clades associated with distinct mosquito genera [70–72], Aedes flavivirus, which is phylogenetically distinct from related Culex flaviviruses, has previously also been found in *Cx*. *pipiens* mosquitoes sampled in Italy [73]. Our findings therefore support not only the vertical transmission of ISFVs in mosquitoes, but also the potential of occasional horizontal transmission between mosquito species and genera. Therefore, ISFVs in mosquito populations represent a promising model for the study of the evolution of host specificity of flavivirus infectivity [72].

Some ISFVs (Palm Creek flavivirus and Culex flavivirus) have been found to inhibit replication of West Nile and Murray Valley encephalitis viruses in the *Ae*. *albopictus* C6/36 cell line and in *Cx*. *pipiens* mosquitoes [65,66]. In contrast, CFAV, also identified in this study, has recently been found to increase susceptibility of dengue virus in an *Ae*. *aegypti* cell line (Aa20) [74] and to be inhibited by the *Wolbachia* endosymbiont (*w*MelPop) used for dengue control in *Ae*. *aegypti* mosquitoes [75,76]. Because there is considerable variability in how ISFVs effect arbovirus superinfections, how vertical transmission of ISFVs affects the competence of mosquito populations to transmit arboviruses, either horizontally to vertebrate hosts or vertically to the next generation, remains largely unknown.

We also detected An(g)FVs only in mosquito populations from the Lake Baringo region, despite the more than seven times greater sample size of *An*. *gambiae* tested from the malaria endemic Lake Victoria region. While it is curious that this ISFV was only detected in malaria mosquitoes from regions with relatively low malaria transmission rates [77], they have been previously identified in *An*. *gambiae* and *Anopheles squamosus* mosquitoes from malaria endemic North-Eastern Province [26], and Coastal and Western Provinces of Kenya (Fig 3). Other closely related Anopheles flaviviruses (AnFVs) (Fig 3) have since been reported in anopheline mosquitoes from Australia [71], Liberia and Senegal [78], and Turkey [79] (Fig 3). Furthermore, transcriptionally active *Flavivirus*-derived endogenous viral elements have been identified in *Anopheles minimus* and *Anopheles sinensis* genomes via *in silico* and *in vivo* analyses [80], which suggests a historical presence of ISFVs in anopheline mosquitoes. Though ISFVs may have important implications in the transmission of medically important arboviruses [70], the study of AnFVs has been limited by their inability to replicate in standard *Aedes* cell line cultures, or even in cell lines of heterologous *Anopheles* species [26,71]. Appropriate *Anopheles* cell line cultures for the *in vitro* replication of the AnFVs will have to be established to further study their role in co-infection with other arboviruses, and possibly malaria parasites [26].

We recorded more diverse vector mosquito species and viruses in samples from Homa Bay County (Table 2), which concurs with reports from previous studies around the Lake Victoria basin [5,16,19,81]. Although adult *Aedes* mosquitoes have been sampled in both study areas [5,82], we only sampled *Aedes* spp. larvae from Lake Victoria. In a previous study, we found that many of the suitable larval habitats for *Ae*. *aegypti* sampled in the Lake Victoria region correlated with increased ammonium and phosphate levels, which are key components of commonly used fertilizers [83]. Our larval sampling strategy may have been more favourable for sampling *Aedes* mosquitoes in the Lake Victoria region where agricultural activity is more intensive in comparison to the Lake Baringo region. Though there was an RVF virus outbreak in Baringo County in 2006/2007 and surveillance studies around the area reported possible mosquito vectors [11,84], none of our mosquito samples from Baringo County tested positive for any pathogenic virus.

Our identification of both a pathogenic arbovirus and three ISFVs in larval mosquitoes from both lake basins suggests complex ecologies involved in their circulation and maintenance. Although Omondi et al. [5] did not detect any virus from blood-fed mosquitoes around the Lake Victoria region where we found vertical transmission of Bunyamwera virus, AeFVs, and CFAV, the study found Bunyamwera virus in blood-fed mosquitoes from the Lake Baringo region, where we found no Bunyamwera infected larvae. Though these discrepant findings may be a result of inadequate sample size required to reliably identify specific arboviruses circulating in a region, the conditions for the maintenance of arboviruses by vertical transmission may depend on environmental factors of the mosquito vector’s reproductive environment. Nonetheless, our findings indicate that in the Lake Victoria region environmental context, *An*. *gambiae*, and possibly *Cx*. *univittatus*, can act as a reservoir that can both vertically and horizontally transmit Bunyamwera virus, ISFVs, and possibly other arboviruses. This is important towards understanding how arboviruses are maintained and geographically spread in different ecological contexts and can be used to forecast risks and improve prevention and other vector management strategies to mitigate future outbreaks. Continued arbovirus surveillance in diverse mosquito and other arthropod vector species in the region will help to more accurately identify the most important vectors of arboviruses possibly associated with febrile illnesses, while a better understanding of the role of ISFVs in the vertical transmission of arboviruses may open new control strategies.

Insect-specific flavivirus NS5 gene sequences from twelve mosquito pools were deposited into the GenBank nucleotide database (accessions MG372051- MG372060, MK015647- MK015648).

## Supporting information

S1 Fig Alignment of Bunyamwera sequence obtained from study samples (grey consensus) with GenBank reference sequence (accession KM507344). Yellow = Guanine; Red = Adenine; Blue = Cytosine; Green = Thymine.
(EPS)
